# The plant microbiome in biotechnology: An annotated selection of World Wide Websites relevant to the topics in *microbial biotechnology*

**DOI:** 10.1111/1751-7915.12449

**Published:** 2016-10-20

**Authors:** Lawrence P. Wackett

**Affiliations:** ^1^ Department of Biochemistry, Molecular Biology and BiophysicsBioTechnology InstituteUniversity of MinnesotaSt PaulMN55108USA

## Biotechnology through understanding plant–microbiome interactions

### http://www.scielo.cl/pdf/jsspn/v15n2/aop2115.pdf

This review describes plant microbiomes and then addresses whether they can be engineered to encourage beneficial organisms, and discourage undesirable ones.

## Agricultural microbiology: Microbe Wiki

### https://microbewiki.kenyon.edu/index.php/Agricultural_microbiology

This Microbe Wiki page deals broadly with plant‐associated microorganisms. It contains some useful references at the end.

## Networking in the plant microbiome

### http://journals.plos.org/plosbiology/article?id=10.1371/journal.pbio.1002378

This open source research article begins to address the complex issue of hub species and how microbiome composition influences plant growth and health.

## Disentangling the plant microbiome

### https://www.sciencedaily.com/releases/2016/07/160712130223.htm

This news‐type article highlights the complexity of plant microbiomes and suggests that engineering them for beneficial traits will likely prove to be more complex than many people currently suggest.

## Indigo mapping plant microbiome

### https://techcrunch.com/2016/02/18/indigo-is-mapping-plant-biomes-to-produce-next-generation-crops/

This news article discusses Indigo, a relatively new company using the knowledge from metagenomic sequencing to help produce microbial seed coatings that deliver beneficial properties.

## Assembling plant microbiomes

### http://jgi.doe.gov/assembling-plant-microbiomes/

This project at the Joint Genome Institute describes a project to connect plant genotypes and phenotypes to the composition of their microbiomes.

## The plant microbiome at work

### http://apsjournals.apsnet.org/doi/abs/10.1094/MPMI-10-14-0334-FI

This article reviews plant microbiome research and makes some suggestions for translating findings to applications in crop science.

## Plant microbiome to improve farming

### http://www.science20.com/the_conversation/understanding_plant_microbiome_can_improve_farming_and_plant_health-152560

This article discusses potential benefits of plant microbiome research that can lead to adding beneficial bacteria or fungi via seed coatings, sprayings, or mixing into mulches.

## Plant microbiome blueprints

### http://science.sciencemag.org/content/349/6250/788

This commentary links to a report in the journal *Science* suggesting that plants play a significant role in organizing the microbiome associated with the plant.

## Phytobiomes organization

### http://www.phytobiomes.org/activities/Pages/meetings.aspx

The phytobiome association provides links for conferences, funding opportunities, and job postings related to plant microbiome research.

## Microbial hub taxa and plant microbiome variation

### https://www.mpipz.mpg.de/4190892/agler_plos_biol_2016_OA.pdf

This research suggests that plant genotype and abiotic factors effect certain “hub” species within the microbiome and that those hubs networks are critical for maintaining community structure.

## Root surface as a frontier for microbiome research

### http://www.pnas.org/content/112/8/2299.full.pdf

This review provides an excellent overview of recent research focused on the root‐microbe interface.

## Root‐microbiome engineering

### https://www.eurekalert.org/pub_releases/2015-09/cp-rme091815.php

In this study, the researchers transplanted microbiomes between plants and showed enhanced growth. The study was conducted with *Arabidopsis*.

## Microbiomes of sugar cane and sphagnum moss

### http://www.joelkostka.net/research/plant_microbiomes/plant_microbiomes.html

This personal website describes research on microbiomes of sugarcane and peat moss. The former is an important agricultural crop and the latter is important in global carbon cycling.

## Plant microbiomes: *C&N News*

### http://cen.acs.org/articles/90/i40/Plant-Microbiomes-Unfurl.html

This *C&E News* article provides a lot of useful information on plant microbiomes with many links to personal websites of scientists in the field and other information.

## AgBiome

### http://agbiome.com

AgBiome is a start‐up in the plant microbiome field striving to create new products derived from biologicals discovered in the plant rhizosphere.

## Augmenting the plant microbiome

### http://www.genomecanada.ca/en/augmenting-plant-microbiome-improve-crop-yield-and-stress-resilience-0

This is a web page derived from the Genome Canada initiative that is dedicated to using genomics to improve crop yield, resistance to heat stress and water efficiency.

## Wageningen plant microbiome network

### http://www.sense.nl/courses/upcoming/10871725/Wageningen-Plant-Microbiome-Network

This organization in the Netherlands is directed towards promoting research on plant microbiomes.

## Why it is time to map the microbiome

### http://www.livescience.com/52962-mapping-the-microbes-in-our-guts-and-the-environment.html

This interesting series of interviews makes the case broadly for microbial metagenomic research, including research into soil environments.

## Rice microbiome: NSF‐funded project

### https://www.nsf.gov/awardsearch/showAward?AWD_ID=1444974

This study will investigate the microbial metagenome in and around rice plants. It will also involve transcriptomic studies.

## US national microbiome initiative

### https://www.whitehouse.gov/sites/whitehouse.gov/files/documents/OSTP%20National%20Microbiome%20Initiative%20Fact%20Sheet.pdf

This United States government initiative details plans to support microbiome research, including that for agricultural advancement. It describes plans for foundation and government agency financial support.

## Monsant microbials

### http://www.monsantobioag.com/global/us/Pages/default.aspx?gclid=CJzYkduZvc8CFQoDaQodR4gH8w

This commercial site contains significant useful information with a resource library, videos, etc.

## Plant and microbes: Discerning friend from foe

### http://www.hhmi.org/research/plant-interactions-microbes-discerning-friend-foe

This personal site describes research on the ability of plant immune systems to discriminate between helpful and harmful bacteria in their environment.

## Marine plant microbiomes

### https://www.mendeley.com/groups/3429591/marine-plant-microbiomes/

This site has a collection of 726 papers as of this date related to the topic. This is a good place to start for references if interested in this topic.

## The plant microbiome in photos: Pinterest

### https://www.pinterest.com/kaleklett/the-plant-microbiome/

Pinterest holds a large collection of photographs and has photos related to plant roots, mycorrhizal fungi and other root–microbe associations.

